# Gestational Bisphenol A Exposure Impacts Embryonic Hypothalamic Microglia Numbers, Ramification, and Phagocytic Cups

**DOI:** 10.3389/fnins.2022.830399

**Published:** 2022-02-18

**Authors:** Jessica M. Rosin, Nikol Tretiakov, Emily Hanniman, Kiana Hampton, Deborah M. Kurrasch

**Affiliations:** ^1^Department of Medical Genetics, Cumming School of Medicine, University of Calgary, Calgary, AB, Canada; ^2^Alberta Children’s Hospital Research Institute, University of Calgary, Calgary, AB, Canada; ^3^Hotchkiss Brain Institute, University of Calgary, Calgary, AB, Canada; ^4^Department of Oral Biological and Medical Sciences, Faculty of Dentistry, University of British Columbia, Vancouver, BC, Canada

**Keywords:** BPA (bisphenol A), microglia, hypothalamus, embryogenesis, phagocytic cups

## Abstract

Microglia are a resident population of phagocytic immune cells that reside within the central nervous system (CNS). During gestation, they are highly sensitive to their surrounding environment and can alter their physiology to respond to perceived neural insults, potentially leading to adverse influences on nearby neural progenitors. Given that bisphenol A (BPA) itself can impact developing brains, and that microglia express estrogen receptors to which BPA can bind, here we asked whether fetal microglia are responsive to gestational BPA exposure. Accordingly, we exposed pregnant dams to control or 50 mg of BPA per kg diet during gestation to investigate the impact of maternal BPA on embryonic hypothalamic microglia. Gestational BPA exposure from embryonic day 0.5 (E0.5) to E15.5 resulted in a significant increase in the number of microglia present in the hypothalamus of both male and female embryos. Staining for microglial activation using CD68 showed no change between control and prenatal BPA-exposed microglia, regardless of sex. Similarly, analysis of cultured embryonic brains demonstrated that gestational BPA exposure failed to change the secretion of cytokines or chemokines, regardless of embryo sex or the dose (50 μg of BPA per kg or 50 mg of BPA per kg maternal diet) of BPA treatment. In contrast, live-cell imaging of microglia dynamics in E15.5 control and gestationally-exposed BPA hypothalamic slices showed increased ramification of microglia exposed to BPA. Moreover, live-cell imaging also revealed a significant increase in the number of microglial phagocytic cups visible following exposure to gestational BPA. Together, these results suggest that gestational BPA exposure impacts embryonic hypothalamic microglia, perhaps leading them to alter their interactions with developing neural programs.

## Introduction

Microglia are a resident population of macrophages and phagocytic immune cells within the CNS. After microglia enter the CNS at embryonic day 9.5 (E9.3), they undergo maturation and begin to survey the parenchyma (Nimmerjahn et al., 2005; Ginhoux et al., 2010; Schulz et al., 2012; Kierdorf et al., 2013; Gomez Perdiguero et al., 2015; Hoeffel et al., 2015; Bennett et al., 2018). During this time, microglia are involved in bidirectional communication with their local environment, which involves unique signaling pathways that allow them to process and respond to dynamic cues that also aid in their maturation (Butovsky et al., 2014; Lavin et al., 2014; Buttgereit et al., 2016; Matcovitch-Natan et al., 2016). During embryogenesis, microglia play unique roles in the development of specific brain regions, including neural progenitor maintenance in the cortex (Antony et al., 2011) and cortical progenitor engulfment as neurogenesis terminates (Cunningham et al., 2013). These important roles for microglia in neurodevelopment become particularly evident in cases of maternal challenge, whereby gestational insults such as stress, infection, and air pollution alter microglial phenotypes (Frank et al., 2010; Levesque et al., 2011, 2013; Bolton et al., 2014; Rosin et al., 2021b,c). Altering microglial physiology in response to environmental stimuli has the potential to adversely affect brain development, since microglia can release pro-inflammatory signals and inappropriately interact with nearby cells, which can drive altered circuitry formation and result in long-term behavioral alterations. For example, maternal cold exposure—a paradigm for maternal stress—elicits a pro-inflammatory response and disrupts oxytocin neuron numbers in the paraventricular nucleus of the hypothalamus only in male embryos (Rosin et al., 2021c). Moreover, these sexually dimorphic disruptions require microglia and continue into adulthood, with cold stress exposed male offspring showing aberrant social deficits (Rosin et al., 2021c). These types of analyses aimed at the long-term consequences of maternal challenge and gestational exposure to environmental stimuli suggest that immature microglia play an unappreciated role in sensing embryonic perturbations and translating these insults into neurodevelopmental disruptions.

Given the importance of microglia during neurodevelopment, and their responsiveness to environmental stimuli, recent research has examined how microglia react to the plasticizer and endocrine disrupting chemical (EDC) bisphenol A (BPA). BPA is commonly used in consumer products, such as plastic bottles and thermal receipts, and can cross the placenta and embryonic blood-brain barrier during gestation (Schonfelder et al., 2002; Nesan et al., 2021). Since BPA exhibits a range of molecular mechanisms through which it can act, a variety of targets for its actions exist. Depending on the receptor type, BPA can function as either an agonist [i.e., binding to estrogen receptor alpha (ERα) and beta (ERβ) or estrogen-related receptor gamma (ERRγ)] or antagonist [i.e., disrupting androgen receptor (AR) signaling or thyroid hormone receptor alpha (TRα) and beta (TRβ) signaling] to produce its effects on cellular functions (Nesan et al., 2018). BPA is best known as a xenoestrogen that can bind estrogen receptors to alter the transcription of estrogen-related genes and lead to altered signaling (Nesan et al., 2018). Given the expression of estrogen receptors in microglia, these cells might be an unexpected target of BPA (Li et al., 2000; Vegeto et al., 2001).

In zebrafish, embryonic exposure to environmentally relevant levels of BPA (0.0068 μM) drives precocious neurogenesis specifically in the developing hypothalamus and concomitant hyperactivity in these animals later in life (Kinch et al., 2015). This study highlights the potential consequences associated with BPA exposure during a critical period of neurodevelopment and supports further investigation into the potential risks associated with exposure to this EDC. Interestingly, recent work in the mouse exploring the impact of gestational BPA exposure at environmental levels (50 μg of BPA per kg maternal diet) on neurodevelopment also show precocious neurogenesis in the hypothalamus, alongside altered behavioral responses, including hyperactivity, disrupted sociability, and altered circadian activity (Nesan et al., 2021). Similarly, various reports from the CLARITY-BPA consortium study National Toxicology Program (2018) demonstrate that administering BPA to rats at a similar dose drives a variety of hypothalamic-specific BPA effects, including alterations in the volume of the anteroventral periventricular nucleus of the hypothalamus (Arambula et al., 2017) and changes in hypothalamic oxytocin receptor expression (Witchey et al., 2019). Alongside these important findings in rodent models, human analyses have also demonstrated that gestational and earl-life exposure to BPA correlates with various neurodevelopmental disorders, such as autism spectrum disorder (ASD), attention deficit hyperactivity disorder (ADHD), anxiety, and depression (Harley et al., 2013; Tewar et al., 2016).

In terms of microglial responses to BPA, *in vitro* work on microglial BV2 cells (100 nM/L BPA treatment) and *in vivo* analyses in a mouse model studying maternal BPA exposure (50 mg/kg diet) across the perinatal period both show increases in the pro-inflammatory cytokines tumor necrosis factor alpha (TNFα) and interleukin 6 (IL-6) (Luo et al., 2014; Zhu et al., 2015). Moreover, the transcriptional and translational levels of ionized calcium-binding adapter molecule-1 (IBA1)—a common microglia marker—were significantly elevated in the prefrontal cortex of female mice exposed to BPA perinatally (Luo et al., 2014). Elevated levels of TNFα and IL-6 have also been observed in the neocortex of mice exposed to BPA (200 μg/kg/day) using maternal oral gavage during the perinatal period, with BPA exposed weanlings displaying increased numbers of amoeboid microglia (Kagawa and Nagao, 2020). Similarly, gestational exposure to BPA (200 μg/kg/day) using maternal oral gavage increases microglia numbers and alters the transcriptional levels of microglial markers (e.g., IBA1) and inflammatory factors (e.g., TNFα), in both the dorsal telencephalon and hypothalamus (Takahashi et al., 2018). Moreover, the involvement of estrogen receptors in microglial responses to BPA is supported by *in vitro* studies using BV2 cells, where treatment with the estrogen antagonist ICI182780 partially reverses the alterations in microglial morphology and inflammatory cytokine secretion observed following BPA exposure (Zhu et al., 2015).

Sex differences have also been reported in studies examining the impact of perinatal BPA exposure on microglia, whereby a significant increase in microglia numbers can be detected in the female adolescent rat prefrontal cortex following BPA exposure at a dose of 40 μg/kg/day, while males displayed a significant decrease in microglia numbers at a dose of 4 μg/kg/day BPA (Wise et al., 2016). Similar sex differences have been reported in prairie voles, where postnatal exposure to BPA at 50 mg/kg body weight significantly elevated microglia numbers in the dentate gyrus of the hippocampus and the posterodorsal portion of the amygdaloid nucleus (MePD) in females, while postnatal BPA exposure at a dose of 5 μg/kg body weight significantly increased male microglia numbers across the amygdala, including the MePD and the anterior portion of the basolateral amygdaloid nucleus (BLA) (Rebuli et al., 2016). Moreover, considering the widespread use of BPA in consumer products and the possible sex-specific effects of BPA, correlative studies on human populations aimed at examining the long-term impacts of BPA exposure on behavior have also been performed. Intriguingly, elevations in urinary BPA concentrations, obtained from pregnant women at 16 and 26 weeks of gestation and birth, were associated with higher scores for measures of anxiety, hyperactivity, emotional control, and behavioral inhibition in children at 3 years of age, whereby these behavioral disruptions were more pronounced in girls (Braun et al., 2011). Although strong evidence shows that microglia react to BPA, microglial responses appear to depend on the brain region, developmental period of exposure, and sex—with microglial susceptibility also being variable depending on the dose of BPA. However, what is still lacking is evidence for how changes in microglia numbers and/or activation signature that result from BPA exposure impact microglial dynamics.

Here, we exposed pregnant mice to control or BPA-laced chow (50 mg of BPA per kg diet; ∼50x higher than US FDA safe exposure levels) during gestation from E0.5 until sample collection at E15.5 and studied the impact of BPA on embryonic hypothalamic microglia. A BPA dose above regulatory levels (50 mg of BPA per kg diet) was selected for the majority of our experimental manipulations because: (1) our previous analyses demonstrated almost identical neurogenic and behavioral results following gestational exposure to either the low (50 μg of BPA per kg diet) or high dose (50 mg of BPA per kg diet) BPA diet (Nesan et al., 2021), consistent with a non-monotonic dose-response relationship of BPA, and (2) other maternal BPA mouse studies that demonstrate altered microglia phenotypes use a BPA dosage of 50 mg/kg diet (Luo et al., 2014). Gestational BPA exposure significantly elevated embryonic microglia numbers in the hypothalamus of both male and female embryos—with no observable changes in activation status based on cluster of differentiation 68 (CD68) staining. Since several studies have reported elevations in pro-inflammatory cytokines and chemokines in response to BPA exposure (Luo et al., 2014; Zhu et al., 2015; Takahashi et al., 2018; Kagawa and Nagao, 2020), we decided to compare both low dose (50 μg of BPA per kg diet; ∼20-fold lower than US FDA safe levels) to high dose (50 mg of BPA per kg diet) BPA exposure across gestation (E0.5–E15.5) in our ELISA analyses, which showed no impact on cytokine or chemokine secretion in males or females. In contrast, analyzing microglial dynamics in E15.5 control and the higher dose BPA exposed hypothalamic slices using live-cell imaging demonstrated that BPA significantly increases microglial ramification and phagocytic cup numbers. These results suggest that gestational BPA exposure may drive precocious development of embryonic hypothalamic microglia, based on elevated microglia numbers, expanded microglial process ramification, and increased numbers of microglial phagocytic cups.

## Methods

### Mouse Handling and Embryonic Sample Generation

**FIGURE 1:**
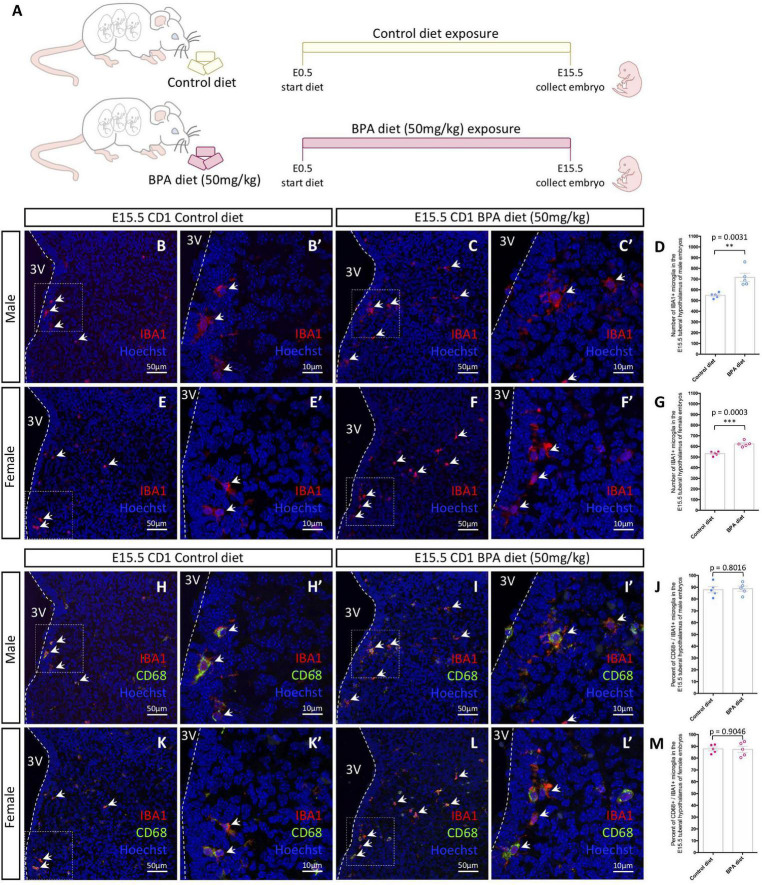
Embryonic hypothalamic microglia are responsive to gestational BPA exposure. **(A)** Schematic diagraming gestational BPA exposure paradigm. **(B–C′)** IBA1 and Hoechst staining in the E15.5 tuberal hypothalamus of control **(B,B′)** and BPA exposed **(C,C′)** male embryos at low **(B,C)** and high **(B′,C′)** magnification. **(D)** Quantification of the number of IBA1 + microglia in the E15.5 tuberal hypothalamus of control and BPA exposed male embryos (*n* = 5; *p* = 0.0031). **(E–F′)** IBA1 and Hoechst staining in the E15.5 tuberal hypothalamus of control **(E,E′)** and BPA exposed **(F,F′)** female embryos at low **(E,F)** and high **(E′,F′)** magnification. **(G)** Quantification of the number of IBA1 + microglia in the E15.5 tuberal hypothalamus of control and BPA exposed female embryos (*n* = 5; *p* = 0.0003). **(H–I′)** IBA1, CD68, and Hoechst staining in the E15.5 tuberal hypothalamus of control **(H,H′)** and BPA exposed **(I,I′)** male embryos at low **(H,I)** and high **(H′,I′)** magnification. **(J)** Quantification of the percent CD68/IBA1 double-positive microglia in the E15.5 tuberal hypothalamus of control and BPA exposed male embryos (*n* = 5; *p* = 0.8016). **(K–L′)** IBA1, CD68, and Hoechst staining in the E15.5 tuberal hypothalamus of control **(K,K′)** and BPA exposed **(L,L′)** female embryos at low **(K,L)** and high **(K′,L′)** magnification. **(M)** Quantification of the percent CD68/IBA1 double-positive microglia in the E15.5 tuberal hypothalamus of control and BPA exposed female embryos (*n* = 5; *p* = 0.9046). **(B–C′,E–F′,H–I′,K–L′)** White arrows point to microglia. White dashed lines mark the third ventricle (3V). **(D,G,J,M)** Counts represent means ± SEM and were analyzed by a Student’s *t*-test. ^**^p < 0.01, ^***^p < 0.001.

**FIGURE 2:**
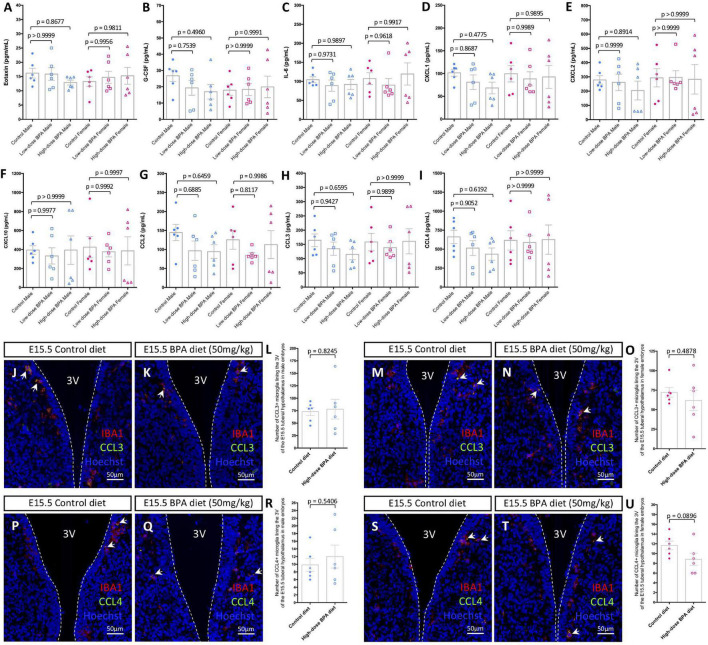
Gestational BPA exposure does not impact cytokine or chemokine secretion. **(A–I)** Quantification of Eotaxin **(A)**, G-CSF **(B)**, IL-6 **(C)**, CXCL1 **(D)**, CXCL2 **(E)**, CXCL10 **(F)**, CCL2 **(G)**, CCL3 **(H)**, and CCL4 **(I)** secretion levels (pg/mL) from cultured control, low dose (50 μg/kg), and high dose (50 mg/kg) BPA exposed male and female brains (*n* = 6). **(J,K)** IBA1, CCL3, and Hoechst staining in the E15.5 tuberal hypothalamus of control **(J)** and BPA exposed **(K)** male embryos. **(L)** Quantification of the number of CCL3/IBA1 double-positive microglia in the E15.5 tuberal hypothalamus of control and BPA exposed male embryos (*n* = 6; *p* = 0.8245). **(M,N)** IBA1, CCL3, and Hoechst staining in the E15.5 tuberal hypothalamus of control **(M)** and BPA exposed **(N)** female embryos. **(O)** Quantification of the number of CCL3/IBA1 double-positive microglia in the E15.5 tuberal hypothalamus of control and BPA exposed female embryos (*n* = 6; *p* = 0.4878). **(P,Q)** IBA1, CCL4, and Hoechst staining in the E15.5 tuberal hypothalamus of control **(P)** and BPA exposed **(Q)** male embryos. **(R)** Quantification of the number of CCL4/IBA1 double-positive microglia in the E15.5 tuberal hypothalamus of control and BPA exposed male embryos (*n* = 6; *p* = 0.5406). **(S,T)** IBA1, CCL4, and Hoechst staining in the E15.5 tuberal hypothalamus of control **(S)** and BPA exposed **(T)** female embryos. **(U)** Quantification of the number of CCL4/IBA1 double-positive microglia in the E15.5 tuberal hypothalamus of control and BPA exposed female embryos (*n* = 6; *p* = 0.0896). **(J,K,M,N,P,Q,S,T)** White arrows point to microglia. White dashed lines mark the third ventricle (3V). **(A–I,L,O,R,U)** Counts represent means ± SEM and were analyzed by a two-way ANOVA with Tukey’s *post hoc* analysis **(A–I)** or by a Student’s *t*-test **(L,O,R,U)**.

**FIGURE 3:**
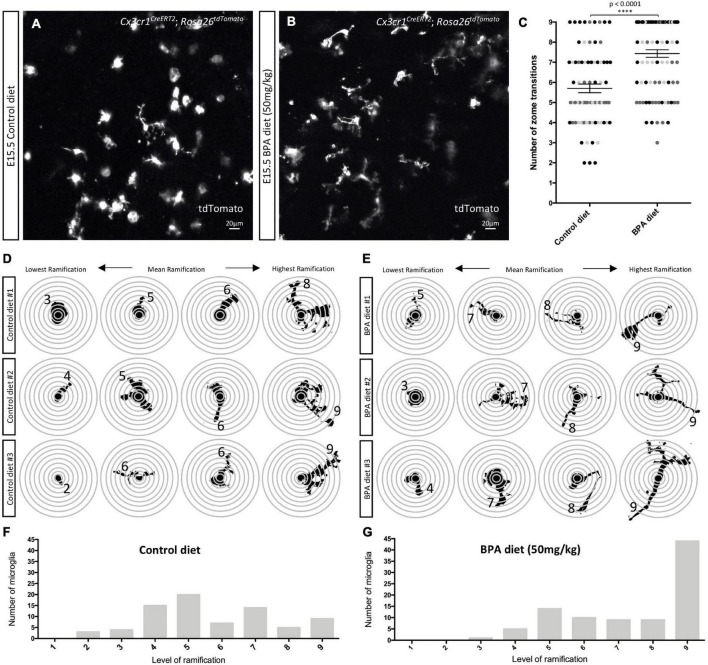
Prenatal BPA exposure enhances microglial ramification in the hypothalamus during embryogenesis. **(A,B)** Stills taken from live-cell imaging videos of E15.5 control and high dose BPA exposed *Cx3cr1^CreERT^*^2^; *Rosa26^tdTomato^* double transgenic hypothalamic brain slices, where microglia are labeled with tdTomato. **(C)** Quantification of the highest level of ramification, displayed as “number of zone transitions,” observed across the entire 5-h live-cell imaging video for each E15.5 control and high dose BPA exposed microglia in *Cx3cr1^CreERT^*^2^; *Rosa26^tdTomato^* double transgenic hypothalamic brain slices (*n* = 3; *p* < 0.0001). Dots that are the same color represent individual microglia from the same embryo/video, while different colored dots represent microglia from different embryos/videos. **(D,E)** Visual depiction of SHOLL analysis performed on microglia from each of the control (**D**; *n* = 3) and high dose BPA exposed (**E**; *n* = 3) embryos analyzed highlight the lowest, mean, and highest level of ramification observed in each independent embryo. **(F,G)** Quantification of the total number of microglia with a defined level of ramification (ranging from 1 to 9) documented across 5-h live-cell imaging videos using E15.5 control (**F**; *n* = 3) or high dose BPA exposed (**G**; *n* = 3) *Cx3cr1^CreERT^*^2^; *Rosa26^tdTomato^* double transgenic hypothalamic brain slices. ^****^p < 0.0001.

**FIGURE 4:**
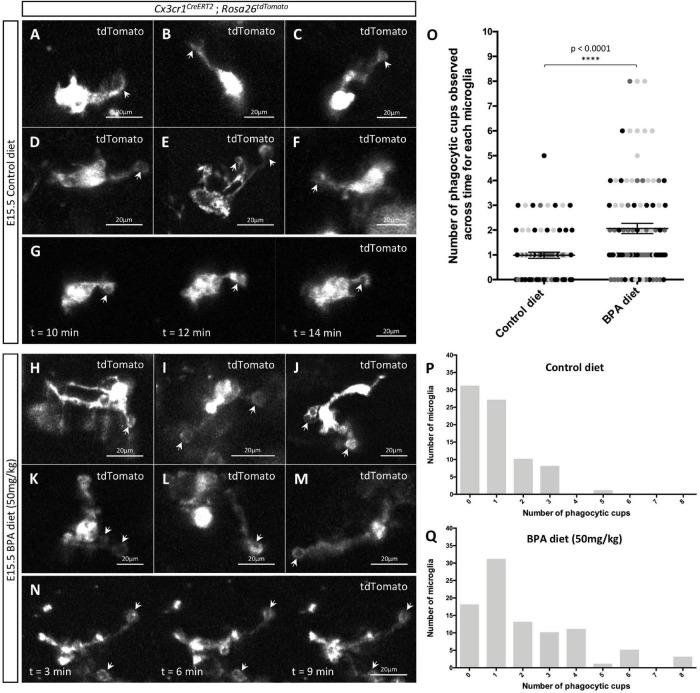
Gestational BPA exposure increases the number of microglial phagocytic cups observable in the hypothalamus during embryogenesis. **(A–N)** Stills taken from live-cell imaging videos of E15.5 control **(A–G)** and high dose BPA exposed **(H–N)** *Cx3cr1*^CreERT^*^2^*; *Rosa26^tdTomato^* double transgenic hypothalamic brain slices, where microglia are labeled with tdTomato and white arrows point to phagocytic cups. **(G,N)** Stills highlighting microglial phagocytic cup activity in control **(G)** and high dose BPA exposed **(N)** embryos across time. **(O)** Quantification of the number of phagocytic cups observed across the entire 5-h live-cell imaging video for each E15.5 control and high dose BPA exposed microglia in *Cx3cr1^CreERT2^*; *Rosa26^tdTomato^* double transgenic hypothalamic brain slices (*n* = 3; *p* < 0.0001). Dots that are the same color represent individual microglia from the same embryo/video, while different colored dots represent microglia from different embryos/videos. **(P,Q)** Quantification of the total number of microglia with a defined number of total phagocytic cups documented across 5-h live-cell imaging videos using E15.5 control (**P**; *n* = 3) or high dose BPA exposed (**Q**; *n* = 3) *Cx3cr1^CreERT2^*; *Rosa26^tdTomato^* double transgenic hypothalamic brain slices. ^****^p < 0.0001.

Animal work was carried out in accordance with the guidelines and regulations of the Canadian Council of Animal Care and received prior approval from the University of Calgary’s Animal Care Committee (protocol AC17-0191). Adult male and female CD1 mice (strain code 022, Charles River) were used to generate the embryonic samples used in Figures 1, 2. Adult male *Cx3crl^CreERT^*^2^ (JAX: 021160) and female *Rosa26^tdTomato^* (JAX: 007914) mice were used to generate the embryonic samples used in Figures 3, 4, where Cre recombinase was induced using 1 mg 4-Hydroxytamoxifen (H7904, Sigma) dissolved in corn oil and administered intraperitoneally to pregnant dams over 2 days (E11.5 and E12.5) as two 0.5 mg doses. Female mice were plug-checked in the morning and those with a vaginal plug were assigned E0.5. During the pregnancy (E0.5 onward) female mice were placed on either a 7% corn oil diet (control diet; Envigo diet code TD.120176), a 50 μg of BPA per kg diet (lower dose BPA; Envigo diet code TD.160491), or a 50 mg of BPA per kg diet (higher dose; Envigo diet code TD.120177) *ad libitum* until E15.5, at which time samples were collected. Prior analyses show that the exposure of BPA to dams is ∼2.25 μg/kg body weight (BW) for the lower dose of BPA (50 μg of BPA per kg maternal diet) and ∼2.25 mg/kg BW for the higher dose of BPA (50 mg of BPA per kg maternal diet) (Nesan et al., 2021). Mice were housed in Techniplast cages with Techniplast water bottles, and our previous analyses demonstrated that serum levels in pups born to dams that ingested the 50 μg/kg BPA diet across gestation had approximately 25-fold higher BPA (∼0.08 ng/ml) than control pups in these same cages (Nesan et al., 2021). Euthanasia, embryo collection, and *Sry* genotyping have been described elsewhere (Rosin et al., 2021c).

### Tissue Preparation and Immunohistochemistry

Tissue preparation and immunohistochemistry protocols have been described previously (Rosin et al., 2021c). In brief, fixed embryonic brains were embedded in Clear Frozen Section Compound (VWR 95057-838) and cryosectioned (10 μm sections, coronal plane) on a Leica Biosystems cryostat. E15.5 cryosectioned brain tissue was placed on Superfrost plus glass micro-slides (VWR 48311-703), where serial sectioned tissue containing the hypothalamus was collected over 8 slides (21 sections per slide) from a single E15.5 brain. Cryosectioned brain tissue was rehydrated, exposed to rabbit anti-FEZF1 to mark the tuberal hypothalamus (1:200, Fitzgerald 70R-7693), rabbit anti-IBA1 (1:500, Wako 019-19741), rat anti-CD68 (1:200, Bio-Rad MCA1957), goat anti-CCL3 (1:200, R&D systems AF450-NA), or goat anti-CCL4 (1:200, R&D systems AF451-NA) at 4°C overnight, followed by secondary antibody incubation and staining with Hoechst 33342 (1:1,000, Life Technologies H3570). Images were captured on a ZEISS LSM 880 with Airyscan confocal microscope and analyzed using ZEN Black software. Brightness and/or contrast of the entire image was adjusted using Adobe Photoshop CC if deemed appropriate.

### Cytokine and Chemokine Assay

Cytokine and chemokine analysis using ELISAs have been described elsewhere (Rosin et al., 2021c). In brief, whole E15.5 brains were extracted from control, lower dose (50 μg/kg), and higher dose (50 mg/kg) BPA exposed CD1 embryos and placed in a 48-well tissue culture plate containing 300 μL of warmed culture media for 6 h in a 37°C incubator with 5% CO_2_. Culture media was collected, spun down at 3,000 g for 10 min at 4°C, and the middle 200 μL of the culture media was removed and flash frozen with liquid nitrogen. The resulting samples were sent to Eve Technologies (Calgary) and analyzed using the Mouse Cytokine Array/Chemokine Array 32-PLEX (MD32).

### Cell Culture and Live-Cell Imaging

E15.5 brains were quickly extracted on ice in cold phosphate-buffered saline (PBS), which was followed by placement in 4% UltraPure LMP agarose (16520050, Invitrogen) in preparation for immediate slicing (350 μm thickness) on a Leica VT1200S Vibratome, as described previously (Rosin et al., 2021a,b). In brief, thick hypothalamic sections were placed on Millicell cell culture inserts (PICM0RG50, Millipore) within a 40 mm glass bottom Petri dish (14026-20, Ted Pella Inc.) containing 1.5 mL of culture media and placed in a 37°C incubator with 5% CO_2_ for 1 h prior to imaging. During the live-cell imaging experiments, the glass bottom Petri dishes containing the hypothalamic slices were placed in a heated chamber at 37°C with 5% CO_2_ and imaging was performed on slices containing the hypothalamic paraventricular nucleus across 5 h on a ZEISS LSM 880 with Airyscan confocal microscope, and the resulting live-cell imaging videos were analyzed using ZEN Black software.

### Quantification and Statistical Analysis

For cell counts, 1 of 8 slides (10 μm serial coronal sections, 21 sections/slide) captured from every embryo was stained with FEZF1 (FEZF1 marks the tuberal hypothalamus). The FEZF1+ sections were used for microglia counts, based on co-staining between IBA1 and Hoechst (Figures 1B–G), activated microglia counts, based on IBA1, CD68, and Hoechst triple-staining (Figures 1H–M), and IBA1, CCL3, or CCL4, and Hoechst triple-staining, which were used to quantify CCL3+ and CCL4+ microglia (Figures 2J–U). To analyze microglia morphology, SHOLL analysis was used (Figure 3), whereby the number of rings that microglial projections intersected were quantified for each microglia across the 5 h live-cell imaging videos. The number of phagocytic cups, identified by their distinct circular morphology, observed across each of the 5 h live-cell imaging videos was quantified for each microglia (Figure 4). Quantitative results (*n* = 3–6 embryos obtained from 3 to 4 separate pregnant dams) are represented by mean scores ± SEM and were analyzed by either a two-tailed unpaired Student’s *t*-test or a two-way ANOVA with Tukey’s *post hoc* analysis in Prism 6 (GraphPad Software).

## Results

### Hypothalamic Microglia Numbers Are Elevated in Embryos Exposed to Bisphenol A During Gestation

In our previous analyses on the impact of BPA exposure on hypothalamic development (Nesan et al., 2021), we established a mouse paradigm for gestational BPA exposure, whereby we characterized two BPA-laced diets: 50 μg of BPA per kg diet (referred to as “low dose”) and 50 mg of BPA per kg diet (our “higher dose”). Interestingly, these analyses demonstrated almost identical neurogenic and behavioral results following gestational exposure to either the low or high dose BPA diet (Nesan et al., 2021). Taking this into account, alongside other maternal BPA mouse studies showing altered microglia phenotypes using 50 mg/kg BPA diet (Luo et al., 2014), we decided to use the 50 mg/kg BPA maternal diet as the main paradigm for our analyses here. To start, pregnant CD1 females were placed on either a control or 50 mg/kg BPA diet the day a plug was detected (E0.5), which continued until sample collection (Figure 1A). To determine the impact of gestational BPA exposure on embryonic hypothalamic microglia numbers, E15.5 control and high dose BPA exposed embryos were dissected, sexed, and their brains were sectioned and stained with IBA1 and CD68. Staining for IBA1 in the E15.5 hypothalamus of control (Figures 1B,B’) and BPA exposed (Figures 1C,C’) male embryos showed a significant elevation in the number of IBA1+ microglia in BPA exposed males (Figure 1D; *p* = 0.0031). Similarly, staining for IBA1 in the E15.5 hypothalamus of control (Figures 1E,E’) and BPA exposed (Figures 1F,F’) female embryos showed a significant increase in IBA1+ microglia numbers in gestationally exposed BPA females (Figure 1G; *p* = 0.0003).

In contrast, co-staining with IBA1 and CD68—a phagosome marker used to analyze microglia activation—in the E15.5 hypothalamus of control (Figures 1H,H’) and BPA exposed (Figures 1I,I’) male embryos did not reveal alterations in the percent of CD68+ microglia following BPA exposure (Figure 1J; *p* = 0.8016). Similarly, IBA1 and CD68 co-staining in the E15.5 hypothalamus of control (Figures 1K,K’) and BPA exposed (Figures 1L,L’) female embryos did not show changes in the percent of CD68+ microglia following gestational BPA exposure (Figure 1M; *p* = 0.9046). Together, these findings suggest that gestational exposure BPA elevates microglia numbers in both male and female embryos, without obvious changes to microglia activation.

### Gestational Bisphenol A Exposure Does Not Alter Pro-Inflammatory Cytokine or Chemokine Secretion During Embryogenesis

Previously, the impact of BPA on microglial pro-inflammatory signaling was studied in the postnatal brain (Luo et al., 2014) and/or focused on changes in transcript levels as a readout for altered signaling (Takahashi et al., 2018; Kagawa and Nagao, 2020). Here, instead we measured cytokine and chemokine levels in conditioned media from *ex vivo* embryonic preparations to inform on “active” signaling alongside embryonic neurodevelopment, as done previously (Rosin et al., 2021c). Whole E15.5 brains were extracted from control, lower dose (50 μg/kg) and higher dose (50 mg/kg) BPA exposed male and female embryos, cultured at 37°C for 6 h and analyzed using an ELISA. Of the 32 cytokines and chemokines assayed, we only detected Eotaxin (Figure 2A), granulocyte colony-stimulating factor (G-CSF; Figure 2B), IL-6 (Figure 2C), chemokine C-X-C motif ligand 1 (CXCL1; Figure 2D), CXCL2 (Figure 2E), CXCL10 (Figure 2F), C-C motif chemokine ligand 2 (CCL2; Figure 2G), CCL3 (Figure 2H), and CCL4 (Figure 2I) levels in our preparations. Surprisingly, we did not observe differences between control and BPA exposed brains for the cytokines and chemokines measured in our assay (Figures 2A–I), regardless of sex or dose (50 μg/kg vs. 50 mg/kg).

Given that previously we identified a unique population of CCL3/CCL4-expressing microglia that reside adjacent to neural stem cells (NSCs) in the embryonic hypothalamus (Rosin et al., 2021c), and that BPA impacts hypothalamic neurogenesis (Kinch et al., 2015; Nesan et al., 2021), we quantified the number of CCL3+ and CCL4+ microglia in the embryonic hypothalamus in BPA exposed embryos. Co-staining with IBA1 and CCL3 in the E15.5 hypothalamus of control (Figure 2J) and BPA exposed (Figure 2K) male embryos did not show alterations in the number CCL3+ microglia following gestational BPA exposure (Figure 2L; *p* = 0.8245). Similarly, co-staining with IBA1 and CCL3 in the E15.5 hypothalamus of control (Figure 2M) and BPA exposed (Figure 2N) female embryos did not display changes in CCL3+ microglia numbers in brains following gestational BPA exposure (Figure 2O; *p* = 0.4878). Co-staining with IBA1 and CCL4 in the E15.5 hypothalamus of control (Figure 2P) and BPA exposed (Figure 2Q) male embryos showed no changes in CCL4+ microglia numbers following gestational BPA exposure (Figure 2R; *p* = 0.5406). Moreover, IBA1 and CCL4 co-staining in the E15.5 hypothalamus of control (Figure 2S) and BPA exposed (Figure 2T) female embryos did not demonstrate altered CCL4+ microglia numbers following BPA exposure (Figure 2U; *p* = 0.0896). Collectively, this data suggests that gestational BPA exposure does not alter cytokine or chemokine secretion during embryogenesis, regardless of sex or the dose of BPA treatment.

### Maternal Bisphenol A Exposure Impacts Embryonic Hypothalamic Microglial Ramification

Previous analyses of fixed tissue from the mouse neocortex of weanlings exposed to BPA across the perinatal period suggest that BPA increases the number of amoeboid microglia (Kagawa and Nagao, 2020). We were interested in characterizing changes in microglial morphology in the embryonic hypothalamus, but instead we visualized the dynamic changes in microglial processes in real time. We employed our previously established live-cell imaging protocol (Rosin et al., 2021a,b) and cultured 350 μm thick sections of the hypothalamus from E15.5 C-X3-C motif chemokine receptor 1 (*Cx3cr1*)*^CreERT^*^2^; *Rosa26^tdTomato^* double-transgenic control and the higher dose BPA exposed embryos to visualize tdTomato+ microglia across a period of 5 h. Considering that BPA did not show sex-specific effects in microglial numbers (Figure 1) or cytokine/chemokine secretion (Figure 2) and given the time sensitive nature of live-cell imaging experiments (i.e., < 1 h to start imaging is insufficient to sex embryos prior to the start), the embryonic samples used for live-cell imaging were not sexed. Although E15.5 control microglia were very dynamic, they appeared to display less ramification as compared to BPA exposed microglia (stills in Figures 3A,B and Supplementary Videos 1, 2).

To quantify microglia morphology, we conducted a SHOLL analysis, whereby the number of rings that microglial projections intersected (i.e., zone transitions) were counted (Figures 3C–G). Quantification of the maximum number of zone transitions for each microglia analyzed across the entire 5-h live-cell imaging video demonstrated that gestational BPA exposure significantly increases microglial ramification as compared to control (Figure 3C; *p* < 0.0001). Moreover, further analysis of microglial process lengths from individual embryos demonstrated that the lowest level of ramification observed in controls ranged from 2 to 3 rings versus 3 to 5 ring intersections in BPA embryos, while mean ramification ranged between 5 and 6 rings in controls as compared to 7 and 8 rings in BPA embryos (Figures 3D,E). Furthermore, there was a clear shift in microglial process length observable following BPA exposure when the total number of microglia with a specific level of ramification (ranging from 1 to 9 rings) was plotted for control (Figure 3F) and higher dose BPA exposed (Figure 3G) embryos. Taken together, these data suggest that gestational BPA exposure enhances embryonic hypothalamic microglia ramification.

### Prenatal Exposure to Bisphenol A Enhances the Appearance of Microglial Phagocytic Cups in the Embryonic Hypothalamus

Microglia remove apoptotic cellular debris through the process of phagocytosis (Parnaik et al., 2000; Neumann et al., 2009; Sierra et al., 2010, 2013). Interestingly, phagocytosis by ramified microglia involves the formation of rounded structures called phagocytic cups (Swanson, 2008; Sierra et al., 2010; Perez-Pouchoulen et al., 2015; VanRyzin et al., 2019), and recent analyses suggest that microglia can use phagocytic cups to remove newly born cells to shape their surrounding environment (VanRyzin et al., 2019). Accordingly, we were interested in whether gestational BPA exposure impacts microglial phagocytic cups during embryogenesis; therefore, we further analyzed our E15.5 *Cx3cr1^CreERT^*^2^; *Rosa26^tdTomato^* hypothalamic live-cell imaging videos from control and higher dose BPA exposed embryos to study microglial phagocytic cups. While we were able to visualize phagocytic cups in both our E15.5 control (Figures 4A–G, arrows and Supplementary Video 1) and BPA exposed (Figures 4H–N, arrows and Supplementary Video 2) hypothalamic slices, quantification of the total number of phagocytic cups visualized across the 5 h live-cell imaging video for each microglia showed a significant increase in phagocytic cup numbers following BPA exposure (Figure 4O; *p* < 0.0001). This shift was further evident when the total number of microglia documented with a specific quantity of phagocytic cups (ranging from 0 to 8 phagocytic cups per microglia) was plotted for controls (Figure 4P) and higher dose BPA (Figure 4Q) embryonic hypothalamic microglia. Together, by visualizing microglial dynamics in real time using live-cell imaging, our data demonstrates that gestational BPA exposure causes increased numbers of microglial phagocytic cups per microglia.

## Discussion

Here, we used a maternal BPA exposure mouse model to study the impact of BPA on embryonic hypothalamic microglia. Our results show that BPA exposure from E0.5 to E15.5 increases microglia numbers in the hypothalamus of both male and female embryos—yet these increases are not associated with changes in microglia activation status as measured by CD68 or alterations in pro-inflammatory cytokine or chemokine secretion. Uniquely, live-cell imaging of microglia dynamics in E15.5 control and BPA exposed hypothalamic brain slices demonstrates that BPA increases microglial ramification and phagocytic cup numbers, which together alongside the increases in microglia numbers, suggest that gestational BPA perturbs microglia and could perhaps be indicative of precocious maturation of microglia in the embryonic hypothalamus.

It is important to highlight that most of our analyses were performed using a dose of 50 mg of BPA per kg maternal diet, with actual BPA exposure to dams ∼2.25 mg/kg BW, which is approximately 50 times higher than US FDA safe exposure levels. We did also explore a lower dose of 50 μg of BPA per kg maternal diet (i.e., ∼2.25 μg/kg BW exposure to the dams), which is approximately 22 times lower than the “safe” human RfD of 50 μg/kg BW (Nesan et al., 2021), for our cytokine and chemokine ELISA analyses that yielded non-significant changes. Our previous study shows that dams exposed to lower dose BPA (50 μg/kg) have BPA serum levels of ∼0.4 ng/mL (Nesan et al., 2021), while human studies show BPA levels in maternal serum can vary considerably and range anywhere between 0.46 and 22 ng/mL (Ikezuki et al., 2002; Schonfelder et al., 2002; Kuroda et al., 2003; Padmanabhan et al., 2008), which is 1.15–55-fold higher than what was measured in lower dose BPA exposed pregnant mice. However, considering we used a higher BPA dose for most of the analyses herein, which exceeds safe human exposure levels, the findings we present are only suggestive of what microglia disruptions might be occurring at lower BPA exposure levels.

Assaying microglial responses to BPA across various exposure paradigms suggests that BPA increases microglia numbers, whether that be in the dorsal telencephalon and hypothalamus in mice (Takahashi et al., 2018) or the hippocampus and amygdala of prairie voles (Rebuli et al., 2016), which is in line with our findings. In contrast, while a number of BPA studies focused on microglia have documented enhanced pro-inflammatory signaling in response to BPA exposure (Luo et al., 2014; Zhu et al., 2015; Takahashi et al., 2018; Kagawa and Nagao, 2020), our findings did not highlight substantial changes in any of the 32 cytokines and chemokines assayed, which includes TNFα and IL-6—two pro-inflammatory cytokines consistently shown to be elevated in prior analyses (Luo et al., 2014; Zhu et al., 2015; Takahashi et al., 2018; Kagawa and Nagao, 2020). It is important to note, however, that our data show large variability between individual embryos exposed to BPA; therefore, it is possible that each embryo displays varying sensitivity to BPA. Inconsistencies between our findings and prior literature, such as those related to pro-inflammatory TNFα and IL-6 levels, could also be the result of a variety of other factors, including the method of exposure, period of exposure, or time-point at which the samples were collected. Specifically, in this study, we exposed pregnant female mice to BPA via their diet, with our period of exposure being E0.5–E15.5 and our samples were collected at E15.5, whereas in other studies, oral gavage was used to administer BPA and female rodents were exposed to BPA at varying times (e.g., prior to, during, and/or after pregnancy up until weaning). Moreover, in most cases samples were collected postnatally (Takahashi et al., 2018; Kagawa and Nagao, 2020). Any of these factors could explain the inconsistencies between datasets, especially considering that oral gavage can be stressful to pregnant dams and fetal microglia are responsive to maternal stress. Indeed, we show that maternal stress alters microglial expression signatures and elevates pro-inflammatory cytokines and chemokines during embryogenesis (Rosin et al., 2021c). Consistency in experimental paradigms is important to better compare across models how BPA impacts pro-inflammatory signaling.

Although few studies have investigated the impact of BPA on microglia morphology, prior literature suggests that BPA increases amoeboid microglia numbers in the murine neocortex of weanlings exposed to BPA perinatally (Kagawa and Nagao, 2020). This amoeboid morphology contrasts with our findings here, which show that gestational BPA exposure does not increase microglia activation, but instead, increases microglial ramification. As discussed above, the differences in our findings could result from variations in our methodology. In the context of the present study, it is also possible that performing SHOLL analyses across a 5-h window using live-cell imaging whereby microglia dynamics could be captured, yielded different findings than those observed in fixed tissue preparations. Interestingly, by analyzing microglial phagocytic cups in real time, we uncovered that BPA may impact microglial phagocytic processes. However, since we did not assess the presence of lysozymes in these microglial cells, altered phagocytic activity by these microglia cannot be concluded at this time and requires further experimental analyses. Moreover, whether this change is indicative of enhanced phagocytosis of apoptotic cellular debris or removal of newly born cells is unclear and requires additional research. Furthermore, considering that these data imply that microglia activity and their interactions with the surrounding environment may change as a result of gestational BPA exposure, future studies should also consider other microglial features such as branching density, branching points and the length of individual branches extending from the cell body, in addition to the proximity of microglia to other cells, including neurons, glia, progenitors, and other microglia.

Contrary to prior literature, our findings suggest that gestational BPA exposure causes elevated microglia numbers, expanded microglial ramification, and increased microglial phagocytic cups. These findings do not eliminate the possibility that neuroinflammatory microglia phenotypes exist in other brain regions or at other developmental time-points, such as the postnatal period. Targeted analyses investigating the impact of BPA on microglia using state-of-the-art technologies, such as single-cell RNA sequencing, may yield important findings related to how microglia activity is altered following BPA exposure. These types of studies could provide additional information regarding how BPA changes microglial interactions with neighboring cells and perhaps even provide insights into the impact of BPA on the developing brain given the profound long-term behavioral disruptions observed as a result of embryonic BPA exposure (Kinch et al., 2015; Nesan et al., 2021). Collectively, our findings here suggest that microglia are a potential target for gestational BPA exposure, which are positioned to perturb nearby neural development that might manifest as adverse behavioral outcomes later in life.

## Data Availability Statement

The original contributions presented in the study are included in the article/Supplementary Material, further inquiries can be directed to the corresponding author/s.

## Ethics Statement

The study involving animals was reviewed and approved by the University of Calgary’s Animal Care Committee (protocol AC17-0191). Animal work was carried out in accordance with the guidelines and regulations of the Canadian Council of Animal Care.

## Author Contributions

EH and KH prepared the data for Figure 1. NT performed the cell quantifications for Figure 1. JR performed all other experiments and prepared the manuscript. JR and DK edited the manuscript. All authors read and approved the final manuscript.

## Conflict of Interest

DK is the co-founder of Path Therapeutics, focused on the development of drugs for rare pediatric epilepsies. The remaining authors declare that the research was conducted in the absence of any commercial or financial relationships that could be construed as a potential conflict of interest.

## Publisher’s Note

All claims expressed in this article are solely those of the authors and do not necessarily represent those of their affiliated organizations, or those of the publisher, the editors and the reviewers. Any product that may be evaluated in this article, or claim that may be made by its manufacturer, is not guaranteed or endorsed by the publisher.

## Supplementary Material

Supplementary Video 1 Live-cell imaging video of control *Cx3cr1**^CreERT^*^2^; *Rosa26^tdTomato^*-labeled microglia. Microglial dynamics, including microglial process activity and phagocytic cups, can be observed in the E15.5 hypothalamic brain slice.

Supplementary Video 2 Live-cell imaging video of high dose (50 mg/kg) BPA exposed *Cx3cr1**^CreERT^*^2^; *Rosa26^tdTomato^*-labeled microglia. Microglial dynamics, including microglial process activity and phagocytic cups, can be observed in the E15.5 hypothalamic brain slice.
